# Relationships of PBMC microRNA expression, plasma viral load, and CD4+ T-cell count in HIV-1-infected elite suppressors and viremic patients

**DOI:** 10.1186/1742-4690-9-5

**Published:** 2012-01-12

**Authors:** Kenneth W Witwer, Andria K Watson, Joel N Blankson, Janice E Clements

**Affiliations:** 1 Department of Molecular and Comparative Pathobiology, The Johns Hopkins University School of Medicine, 733 North Broadway, Baltimore, Maryland, 21025, USA; 2 Department of Medicine, The Johns Hopkins University School of Medicine, 733 North Broadway, Baltimore, Maryland, 21025, USA; 3 Department of Neurology, The Johns Hopkins University School of Medicine, 733 North Broadway, Baltimore, Maryland, 21025, USA; 4 Department of Pathology, The Johns Hopkins University School of Medicine, 733 North Broadway, Baltimore, Maryland, 21025, USA

**Keywords:** human immunodeficiency virus, elite suppressor, microRNA, biomarker, NanoString, TaqMan low-density array, viral load, peripheral blood mononuclear cell, CD4+ T-cell

## Abstract

**Background:**

HIV-1-infected elite controllers or suppressors (ES) maintain undetectable viral loads (< 50 copies/mL) without antiretroviral therapy. The mechanisms of suppression are incompletely understood. Modulation of HIV-1 replication by miRNAs has been reported, but the role of small RNAs in ES is unknown. Using samples from a well-characterized ES cohort, untreated viremic patients, and uninfected controls, we explored the PBMC miRNA profile and probed the relationships of miRNA expression, CD4+ T-cell counts, and viral load.

**Results:**

miRNA profiles, obtained using multiple acquisition, data processing, and analysis methods, distinguished ES and uninfected controls from viremic HIV-1-infected patients. For several miRNAs, however, ES and viremic patients shared similar expression patterns. Differentially expressed miRNAs included those with reported roles in HIV-1 latency (miR-29 family members, miRs -125b and -150). Others, such as miR-31 and miR-31*, had no previously reported connection with HIV-1 infection but were found here to differ significantly with uncontrolled HIV-1 replication. Correlations of miRNA expression with CD4+ T-cell count and viral load were found, and we observed that ES with low CD4+ T-cell counts had miRNA profiles more closely related to viremic patients than controls. However, expression patterns indicate that miRNA variability cannot be explained solely by CD4+ T-cell variation.

**Conclusions:**

The intimate involvement of miRNAs in disease processes is underscored by connections of miRNA expression with the HIV disease clinical parameters of CD4 count and plasma viral load. However, miRNA profile changes are not explained completely by these variables. Significant declines of miRs-125b and -150, among others, in both ES and viremic patients indicate the persistence of host miRNA responses or ongoing effects of infection despite viral suppression by ES. We found no negative correlations with viral load in viremic patients, not even those that have been reported to silence HIV-1 in vitro, suggesting that the effects of these miRNAs are exerted in a focused, cell-type-specific manner. Finally, the observation that some ES with low CD4 counts were consistently related to viremic patients suggests that miRNAs may serve as biomarkers for risk of disease progression even in the presence of viral suppression.

## Background

Untreated HIV-1 infection is characterized by viral replication and leads to suppression of the immune system and eventual death in most cases. A minority of individuals, known as elite controllers or elite suppressors (ES), are able in the absence of antiretroviral treatment to resist progression to HIV disease and to maintain peripheral blood viral loads below the limit of detection of current clinical assays (50 copies/mL). The mechanisms whereby this control is achieved remain incompletely understood despite substantial advances in recent years in the characterization of both host and viral factors associated with elite suppression [1-3]. Because a deeper understanding of this phenomenon may facilitate exploitation of the underlying mechanism(s) for vaccine and therapy design, we initiated studies of elite suppression in a relatively new area: the epigenetic realm of small RNA [4].

To date, associations of host microRNA (miRNA) expression with HIV-1 elite suppression have not been described in the literature; however, there has been a strong focus on miRNA in cancer research and, increasingly, in investigations of infectious disease. miRNAs associated with medical conditions may serve as valuable biomarkers [5], especially when present and detectable in easily accessed body fluids, and clinical assays are currently under development for diagnosing and staging specific cancers or monitoring response to treatment. We recently reported that plasma miRNAs upregulated during acute phase infection predict progression in a model of HIV-associated central nervous system disease [6]. The role of miRNA in disease is not restricted to that of bystander or simple consequence of disease processes. miRNAs are often directly involved in etiology and/or response, opening the door to miRNA-based therapies [7,8]. In Hepatitis C virus infection, the liver-enriched miR-122 is required by the virus for optimal replication [9,10]. Inhibiting the action of miR-122 appears to be a viable therapeutic option [11], with an inhibitor currently in phase II trials.

Although the role of miRNA in elite control of HIV-1 is unexplored, multiple studies have examined miRNAs in HIV infection [12]. Several groups have examined the possibility that HIV, like some other viruses, encodes its own miRNAs, which could be processed by cellular machinery [13,14]. Others have probed the effects of HIV on miRNA expression in cell lines or primary cells infected or transfected with HIV [15-19]. Five miRNAs--miRs -28, -125b, -150, -223, and -382--have been implicated in regulation of HIV-1 transcripts through the viral UTR in CD4+ T cells [15]. Other studies have explored the potential role of these miRNAs in the differing susceptibility to infection of monocytes and macrophages [20,21]. Regulatory networks of HIV, miRNAs, antiviral proteins, and miRNA processing enzymes have also been probed [16,22-24]. Importantly, recent work has investigated the *in vivo *expression and roles of miRNAs during lentiviral infection: in peripheral blood mononuclear cells (PBMC) [25], brain tissue [26-28], and plasma [6]. Additional studies and replication will aid the research community in drawing overarching and actionable conclusions.

To investigate the contribution of miRNA-mediated regulation to elite suppression of HIV-1 and to expand our knowledge of HIV and miRNAs in general, we profiled miRNA expression and examined clinical correlates using control, uninfected donors, a well-characterized cohort of elite suppressors, and untreated, viremic HIV-1 infected individuals. We chose to examine PBMC miRNA expression rather than focusing on subsets of cells for several reasons. First, PBMC include the major circulating targets of HIV infection, and knowledge of overall expression is the logical first step towards characterizing cell subtype expression. Second, if an elite suppressor miRNA profile exists and associates with PBMC, isolating cells to measure that profile could be done with standard laboratory equipment and minimal resource commitment. Third, a miRNA profile of infected, viremic PBMC is available for comparison [25]. To maximize confidence in our results and minimize the possibility of platform-specific errors, we used two high-throughput platforms that operate on different principles and also validated the expression of 25 small RNAs using individual quantitative polymerase chain reaction (qPCR) assays [29].

In this study, we show that specific miRNAs distinguish uninfected and elite suppressor PBMC from PBMC of viremic patients. While ES have miRNA profiles that largely cluster with uninfected controls, separation is incomplete, and some ES--specifically, those with the lowest CD4+ T-cell counts--group with viremic patients. Multiple miRNAs are positively or negatively correlated with peripheral blood CD4+ T-cell count, and miRNA expression changes cannot be explained solely by declines in the CD4+ population during infection. Our analysis identifies several miRNAs that have not been previously described in association with HIV infection, including miR-31, which distinguishes controls and ES from viremic individuals and regulates a protein with important implications for T-cell differentiation.

## Results

### NanoString miRNA profiling distinguishes control and ES from viremic individuals

RNA was isolated from peripheral blood mononuclear cells (PBMC) of eight uninfected individuals, seven HIV-1-infected elite suppressors (ES), and seven HIV-1-positive viremic patients (Table 1), using consistent purification technique [30] and simultaneous isolation to avoid batch effects. The NanoString hybridization platform [31] was used to obtain miRNA expression profiles. NanoString uses confocal microscopy to count fluorescently bar-coded probes, detecting and quantitating nucleic acid molecules without amplification or introduction of position-dependent effects. Data were processed using several normalization strategies, including quantile normalization and normalization to a set of invariant miRNAs; such normalization methods have been found to yield consistent and reliable results [32,33].

**Table 1:** ID numbers and characteristics of 22 PBMC donors

ID	Status	CD4	VL	NS	TLDA	qPCR	
						**RT**	**PA**

7	Control			+	+	+	
11	Control			+	+	+	+
15	Control			+	+	+	+
22	Control			+	+	+	+
23	Control			+		+	+
24	Control			+	+	+	+
26	Control			+		+	+
27	Control			+	+	+	+
4 (ES4)	ES	678	< 50	+	+	+	+
5 (ES24)	ES	1491	< 50	+	+	+	+
10 (ES6)	ES	1139	< 50	+	+	+	+
12 (ES3)	ES	1241	< 50	+	+	+	+
16 (ES8)	ES	593	< 50	+	+	+	+
20 (ES25)	ES	572	< 50	+		+	+
21 (ES18)	ES	1762	< 50	+	+	+	+
1	Viremic	383	55990		+		
6	Viremic	326	44440	+	+	+	+
8	Viremic	348	12480	+		+	+
9	Viremic	475	8190	+	+	+	+
13	Viremic	490	155000	+	+	+	+
14	Viremic	213	105660	+	+	+	+
18	Viremic	413	19680	+	+	+	+

Numerical designations are presented along with status (Control, Elite Suppressor, or Viremic), CD4 counts (ES and Viremic), and Viral Load (VL, copies/mL; < 50 = below the limit of clinical assays). Plus signs in the rightmost columns indicate which platforms were used to assay miRNA in each sample: NanoString (NS), TaqMan low-density array (TLDA), and individual quantitative PCR assay (qPCR). The latter was performed with both reverse-transcribed-only (RT) and reverse-transcribed/pre-amplified (PA) material. For ES, two identification codes are given: first, the numbers used in this study to blind samples, and second, the designation used in previous studies using samples from these patients, including [44,55,56].

**Figure 1:**
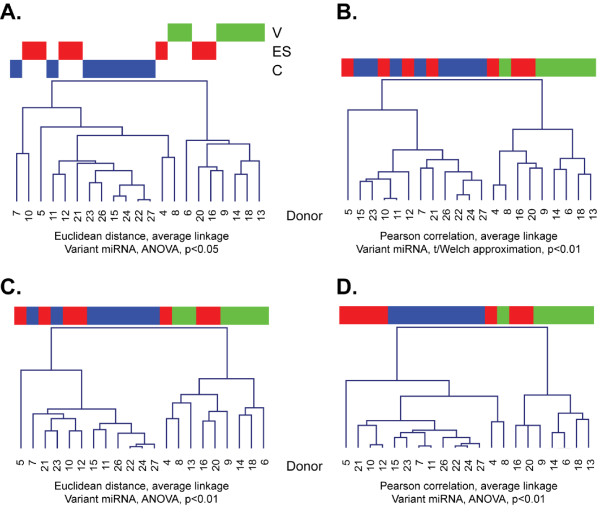
**Hierarchical clustering of NanoString miRNA profiling results**. NanoString miRNA profiling results for PBMC RNA of eight control (C, blue), seven elite suppressor (ES, red), and six viremic (V, green) donors were log-2 transformed and quantile normalized. Values at least one standard deviation above background were included. Data were filtered using one-way analysis of variance (ANOVA, A, C, D) or t-tests with Welch approximation (B). Features with significant difference at p < 0.05 (A, about 50 features) or p < 0.01 (about 20 features, B-D) were used for hierarchical clustering of samples by Euclidean distance or Pearson correlation and average linkage, as indicated. Additional tests using complete linkage were performed with comparable outcome (not shown).

**Figure 2:**
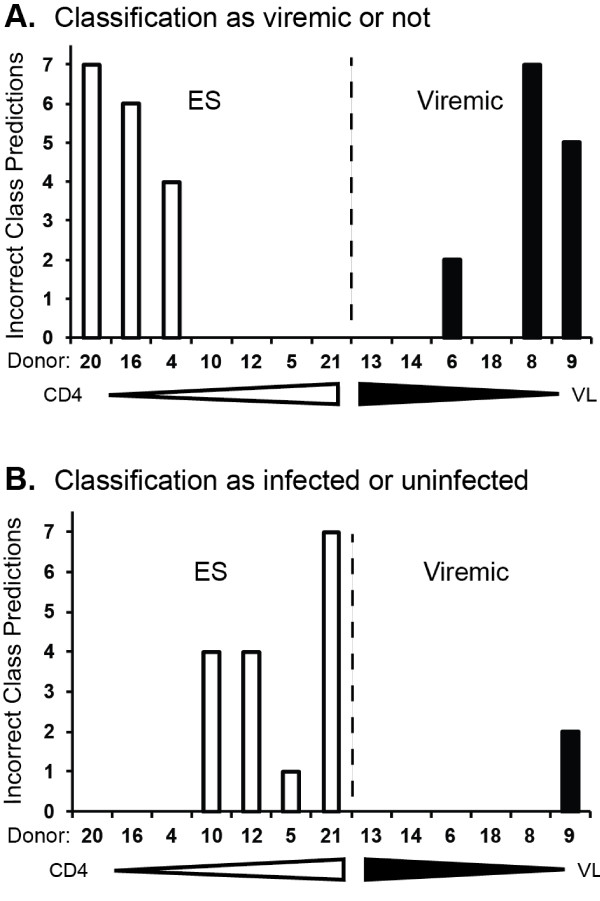
**Incorrect class predictions of ES and viremic samples relate to CD4 count and viral load**. Eight control, seven ES, and six viremic samples were grouped as control+ES vs. viremic (A) and infected vs. uninfected (B). Seven class prediction methods were applied to these groups: Compound covariate, diagonal linear discriminate analysis, 1-nearest neighbor, 3-nearest neighbors, nearest centroid, support vector machines, and Bayesian compound covariate predictor. The number of incorrect class predictions for each ES and viremic sample is shown. Controls are omitted due to a high level of correct predictions for uninfected samples. CD4 count gradients for ES and viral load gradients of viremic samples are indicated with open and filled triangles, respectively (not to scale). ES (open bars) with the lowest CD4 counts were most likely to be misclassified with viremic samples in the comparison of control and ES with viremic samples (A). The class of ES with the highest CD4 counts was most likely to be incorrectly predicted when the two classes were infected and uninfected.

Multiple analyses using two types of clustering revealed that the NanoString PBMC miRNA profiles of viremic patient discriminated them from PBMC of healthy donors; however, the classification of elite suppressors was less facile. By unsupervised hierarchical clustering, ES samples tended to be more related to controls, but several grouped with the viremic samples (Figure 1). This finding was method-independent, as different clustering methods and sets of features led to comparable results (Figure 1A-D). In a second approach, class prediction, we first assigned two classes: the viremic class and a class with undetectable viral loads (control and ES). Seven class prediction methods were used to predict the class of all samples (Figure 2A). Sixteen samples (including all controls, which are not shown) were consistently correctly classified. However, five samples were misclassified by at least four of seven prediction methods (Figure 2A). These samples were elite suppressors 4, 16, and 20 (ES with the lowest CD4+ T-cell counts) and viremic samples 8 and 9, viremic patients with the lowest viral loads (see Table 1). We repeated the analysis with a different grouping: classes of uninfected and infected (ES and viremic). The prediction methods tended to misclassify elite suppressors with high CD4+ T-cell counts as uninfected (Figure 2B; controls again not shown because of consistently correct classification), while viremic samples were predicted correctly. Thus, by miRNA expression, ES samples with high CD4+ T-cell counts appear to relate most closely to PBMC from healthy donors, and viremic samples with relatively low viral loads are more likely to resemble ES or control samples. These results demonstrate an intimate connection of miRNA expression with CD4+ T-cell count and viral load, classical parameters of HIV-1 disease progression.

### Differential expression of individual miRNAs

**Figure 3:**
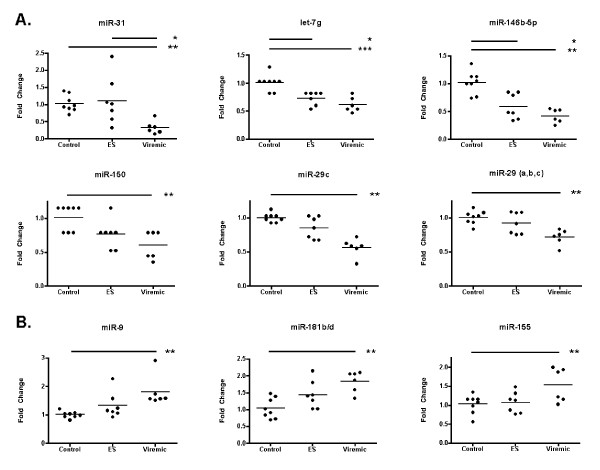
**NanoString profiling indicates differential regulation of specific miRNAs**. Most differentially expressed miRNAs were downregulated in viremic samples (A), although upregulated miRNAs were also observed (B). The final panel in (A) shows the geometric mean of the three miR-29 family members for each sample. Quantile normalized values for miRNA species in each sample were plotted as fold change in comparison with the mean expression of each respective miRNA within the uninfected control group. Significance was assessed by Kruskal-Wallis test followed by Dunn's multiple comparison test, using the absolute distance of normalized expression values from the control mean. Asterisks represent p-values: * = p < 0.05; ** = p < 0.01, *** = p < 0.001.

We next queried the dataset to determine which individual miRNA species were differentially regulated. We observed that among miRNAs with significant expression changes, levels were more often lower in viremic individuals. For example, control PBMC had higher mean levels than viremic PBMC (Figure 3A) of miR-31 (3.9-fold), miR-146b-5p (2.6-fold), miR-125b (2.5-fold), and miR-29c (1.9-fold). Three miRNAs, miRs -9, -155, and -181b/d, increased with infection (Figure 3B). For both up- and downmodulated miRNAs, ES had mean expression that was either intermediate between control and viremic samples or comparable to control levels.

### TLDA profiling confirmed NanoString results and identified additional targets

Many recent studies have examined the sensitivity and specificity of different miRNA quantitation platforms (including but not limited to [34,35]). Between-platform concordance of results is less than perfect, with differences arising from factors including, among others, feature sets that do not overlap completely, including choice of incorporated endogenous controls; platform-specific sample processing requirements; and relative sensitivity and specificity of individual probes and/or primers. Enhanced confidence in results is therefore obtained by using multiple platforms, as we have done in this study.

Because of these considerations and the relative novelty of the NanoString technology, we used Applied Biosystems TaqMan low-density arrays (TLDAs) as a second profiling method. This approach employs reverse transcription, pre-amplification, and a well-established system of quantitative real-time polymerase chain reaction (qPCR) to quantitate even low-abundance miRNAs with high sensitivity. In addition, the TLDAs allowed us to achieve broader coverage of the miRNome. Approximately 550 "features," i.e., miRNAs, small RNAs, or genes, are common to the Applied Biosystems and NanoString platforms. NanoString measures over 100 non-overlapping features, while the TLDAs expand the search space by about 200 miRNAs. We used two TLDA cards each to profile six samples per category (control, ES, and viremic), all but one of which had been assayed by NanoString.

**Figure 4:**
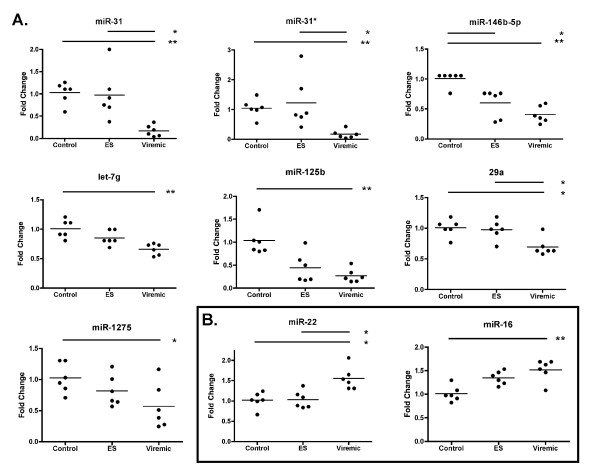
**Individual miRNAs: TaqMan low-density array profiling results**. Downregulated (A) and upregulated (B) miRNAs were identified from quantile-normalized TaqMan low-density array data. Significance was assessed as described for Figure 3. Asterisks represent p values: * = p < 0.05; ** = p < 0.01, *** = p < 0.001.

**Figure 5:**
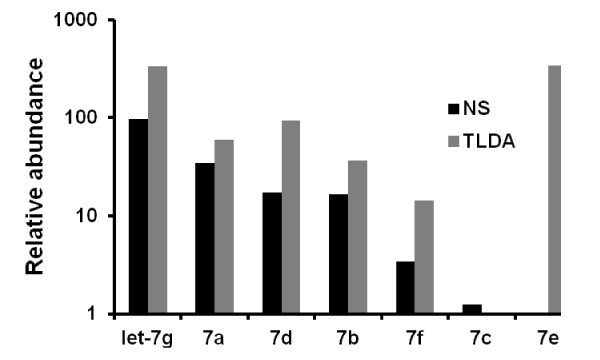
**Relative let-7 family member abundance by NanoString and TLDA**. Relative expression of the indicated let-7 family members (averaged across all samples) was calculated by comparison with one, set equal to expression of the lowest-expressing family member according to each platform: let-7e (NanoString) and let-7c (TLDA).

The TLDA results validated the NanoString findings and also revealed additional candidates for differential expression status (Figure 4). miRNAs with significant downmodulation confirmed by both platforms included miR-31, let-7g, and miR-146b-5p (Figure 4A); TLDA results indicated 10-fold, 1.5- fold, and 2.7-fold downregulation for these miRNAs, respectively. Several miRNAs that ranked highly in the NanoString assessment of differential expression were found by TLDA to have greater fold change expression, a result that may be consistent with a slight compression of data by NanoString for some features as has been reported for hybridization arrays [36]. The TaqMan arrays also identified additional miRNAs as upregulated in viremics and/or ES (Figure 4B), including miRs -16 and -22, or downregulated, such as miR-31* (the hairpin partner of miR-31) and miR-1275. Differential expression of miR-29c (and all miR-29 family members together) was significant by NanoString; in the TLDA results, miR-29a was underexpressed in PBMC from viremics. Whereas let-7e was the most highly expressed let-7 family member in the TLDA dataset, by NanoString it was the least abundant. However, rank order of the other family members was comparable, and let-7g--found by both methods to be differentially regulated--had a similar position in both (Figure 5). These results underscore the advantages of profiling with multiple methods: miRNAs not assayed by one platform (e.g. miR-181b by TLDA or miR-31* by NanoString) may be covered by another, and nonlinear or low-sensitivity assays, which will inevitably be found on any platform, can also be complemented. Most importantly, concordant data bolster confidence in results.

### Validation by individual stem-loop quantitative PCR

**Figure 6:**
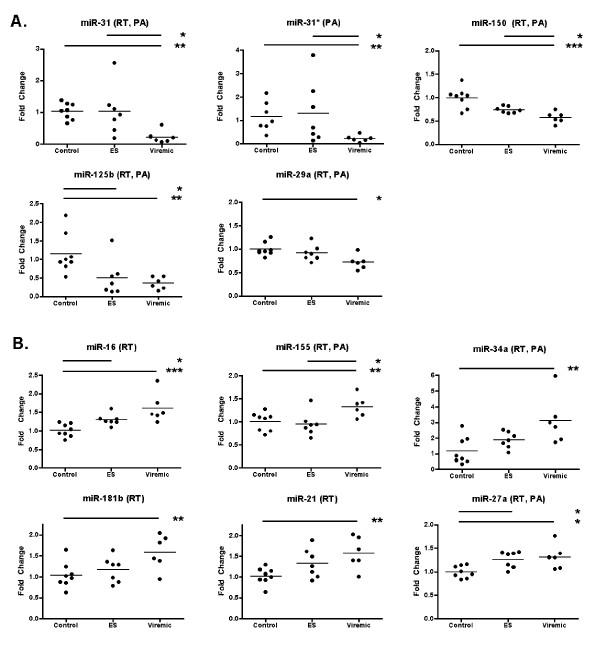
**Individual qPCR miRNA results**. Selected results of individual quantitative PCR assays, with fold change for each sample depicted as compared with the mean of uninfected controls. Samples were normalized (delta-Ct) to the geometric mean of the most invariant twelve RNAs of 23 measured RNA species. Shown are miRNAs downregulated (A) or upregulated (B) in viremic PBMC samples. Asterisks represent p values: * = p < 0.05; ** = p < 0.01, *** = p < 0.001; statistical calculations were done using delta-delta Ct values, not the exponential fold change values. Input materials for each assay were the products of reverse-transcription (RT) or of reverse-transcription and pre-amplification (PA). Where both RT and PA were done, the average of delta-delta Ct values was taken. Three to six replicates were performed for each sample.

**Figure 7:**
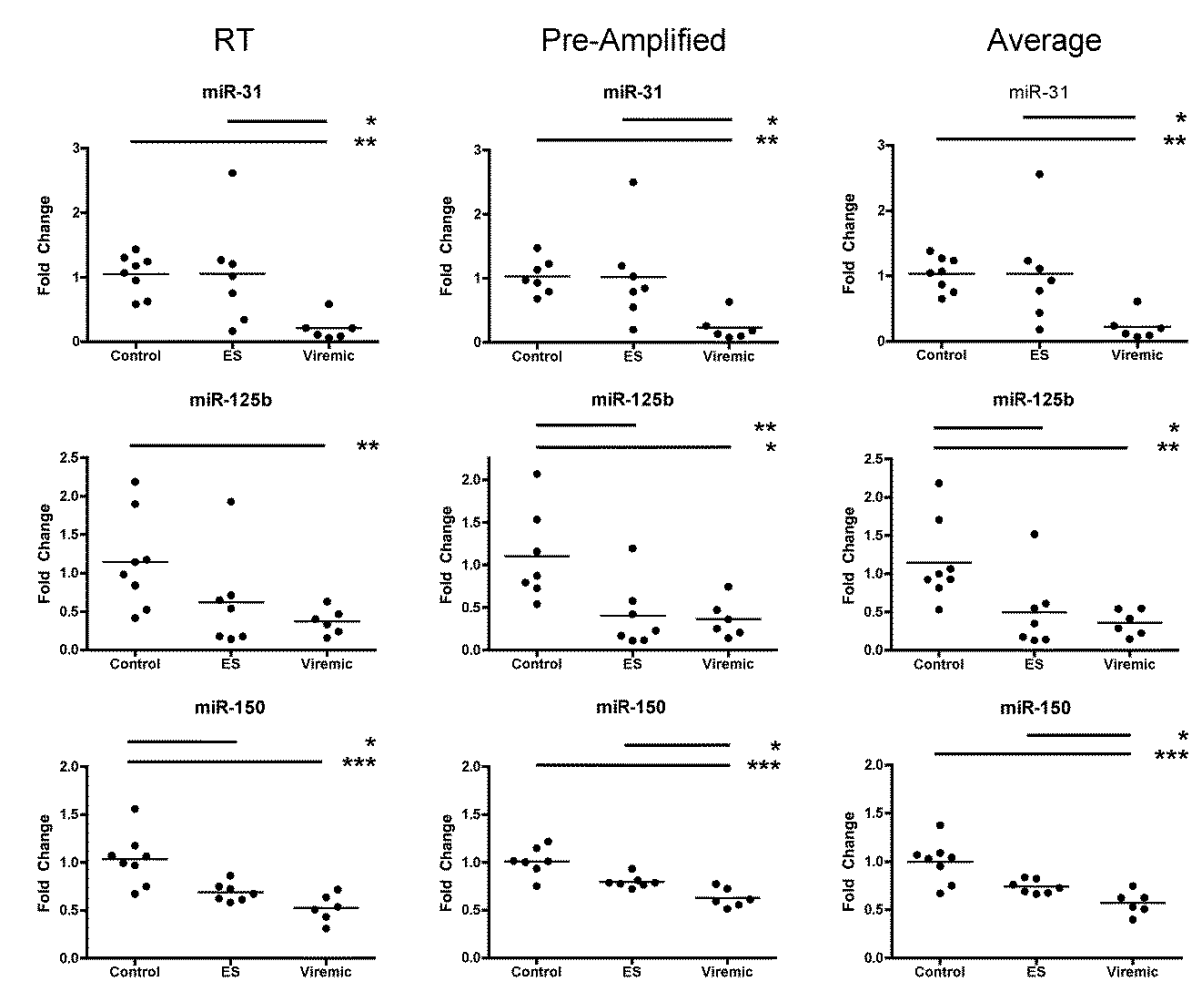
**Comparison of results from qPCR assays with reverse-transcribed or pre-amplified input**. Three representative examples of assays performed with both reverse-transcribed RNA (RT) and reverse-transcribed, then pre-amplified samples. The average, calculated as described in the Figure 7 legend, is also shown. Few substantial differences were observed between assays using samples derived with the two pre-processing variations.

**Figure 8:**
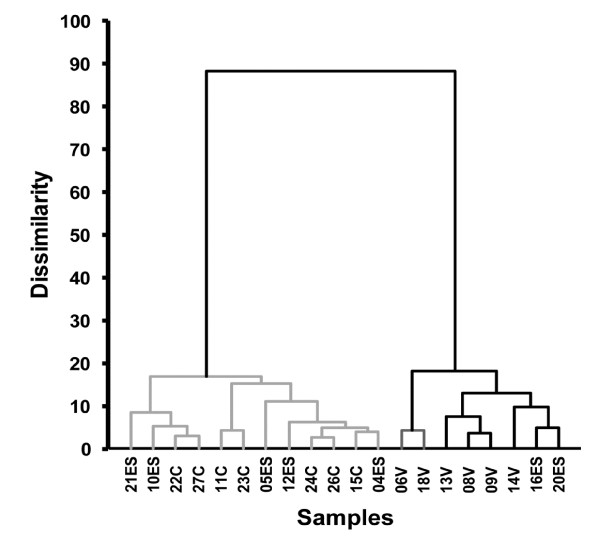
**Agglomerative hierarchical clustering of samples by miRNA expression: individual qPCR assays**. Results of 23 individual small RNA assays were normalized to the geometric mean of the twelve most invariant RNAs and subjected to unsupervised hierarchical clustering. Euclidean distance and complete linkage was used.

To validate our results further, we chose a focused group of more than 25 small RNAs for follow-up by individual quantitative PCR assay. Individual assays differ from those performed in the TaqMan cards in several respects and allow larger numbers of technical replicates to be tested than is feasible with TaqMan arrays. miRNAs were measured in reverse-transcribed samples, reverse transcribed samples that were also pre-amplified, or both (Figure 6). The ten small RNAs quantified in both types of sample were miRs- 27a, -29a, -29c, -31, -34a, -125b, -150, -155, and -221, along with the snRNA U6. Few substantial differences between pre-amplified and unamplified samples were found (for representative comparisons, see Figure 7). The individual miRNA assays confirmed many of the previous results, including the relationship of the three classes by hierarchical clustering and the tendency of ES with low CD4+ T-cell count to cluster with viremic patients (Figure 8). Again, both miR-31 and miR-31* were expressed at significantly higher levels in control and ES PBMC (whether grouped together or analyzed separately) than in cells from viremic patients. Although the elite suppressor miR-31 levels were closer to those of control samples, they also covered a wider range. Levels of miR-181b--elevated according to NanoString but not tested by TaqMan array--were indeed higher in viremic patients than in controls or ES.

### Confidence-based hierarchy of differentially regulated miRNAs

Integrating the results of the three profiling methods, we constructed a list of presumptive differentially expressed miRNAs based on confirmation by independent assays and significance of expression differences of controls and ES from viremic patients (Table 3). We included only those miRNAs that had apparently high expression levels as defined by cycle of amplification by quantitative PCR (Ct < 30) or at least two standard deviations above background (NanoString). The highest levels of confidence, in descending order, are associated with the following ten miRNAs. miR-31, miR-125b, and miR-150 were significantly (p < 0.05) downregulated according to all three profiling methods. miRs -31*, -181b, and -9 were assayed by only two of the three methods, but were significant in both. miR-29a changed significantly (TLDA, qPCR), or trended in the same direction (NanoString) with p < 0.1. For miR-16, miR-146b-5p, and let-7g, significant changes were found by two methods, but not the third.

**Table 2:** Correlations of miRNA expression and CD4+ T-cell count

Negative correlation		Positive correlation	
miRNA	p-value	miRNA	p-value
miR-181b	< 0.002	miR-31	< 0.001
miR-16	0.051	miR-31*	< 0.004
miR-130b	0.051	miR-29a	0.015
miR-155	0.057	miR-150	0.022
		miR-29c	0.054
		miR-342-3p	0.075
		miR-34a **	0.018

For all infected samples and for all miRNAs measured with multiple replicates by quantitative PCR, Pearson product-moment correlation coefficients were calculated and corresponding p-values were determined using XLStat. An alpha of 0.05 was considered significant; additional results that approached significance are also shown. miR-34a (**) was significantly correlated only when the elite suppressors were considered alone.

**Table 3:** Ranking of findings by strength of evidence across platforms

	NanoString		TLDA		qPCR	
miRNA	p	FC	p	FC	p	FC
**miR-31**	**0.0059**	**-3.9**	**0.0026**	**-10.3**	**0.0040**	**-9.2**
**miR-125b**	**0.0419**	**-2.5**	**0.0066**	**-4.5**	**0.0090**	**-3.6**
**miR-150**	***0.0071***	***-1.9***	***0.0261***	***-1.6***	**0.0003**	**-1.6**
miR-31*	**NA**		**0.0043**	**-10.1**	**0.0195**	**-8.5**
**miR-9**	**0.0033**	**1.8**	**0.0489**	**4.1**	**NA**	
**miR-181b**	**0.0092**	**1.8**	**NA**		**0.0400**	**1.6**
*miR-29a*	*< 0.1*		*0.0117*	*-1.5*	*0.0180*	*-1.7*
*let-7g*	*0.0004*	*-1.7*	*0.0028*	*-1.5*	*NS*	
*miR-16*	*NS*		*0.0061*	*1.5*	*0.0001*	*1.5*
*miR-146b-5p*	*0.0025*	*-2.6*	*0.0020*	*-2.7*	*NS*	
miR-155	0.0071	1.5	NS		0.0690	1.3
miR-34a	< 0.1		0.0514	3.6	0.0040	3.1

For NanoString, TaqMan low-density array (TLDA), and quantitative PCR (qPCR), miRNAs are presented according to the estimated strength of evidence for differential regulation between viremic patients and controls: bold (p < 0.05 for each method with which the respective miRNA was measured); italics (p < 0.05 for two of three); and regular font (p < 0.05 for one platform and p < 0.1 for at least one other). Each of the listed miRNAs had relatively high levels of expression across samples: more than two standard deviations above background (NanoString) and/or amplifying before a threshold cycle of 30 by TaqMan assay. Abbreviations: p = p value; FC = mean fold difference from control (positive or negative as indicated); NS (not significant) refers here to p > 0.1; NA: not measured with the indicated method. miR-150 values for NanoString and TLDA are rendered in bold italics to indicate quantile normalization limitations at expression extremes (miR-150 is one of the most abundant miRNAs in PBMC).

### miRNA correlations with viral load

Having established that expression of miRNAs differs significantly across the three sample classes, we wished to discover what relation, if any, these miRNAs have to disease progression. Accordingly, we sought to identify correlations of miRNA expression (individual qPCR assays) with two important clinical parameters of HIV disease: peripheral blood viral load and CD4+ T-cell count.

**Figure 9:**
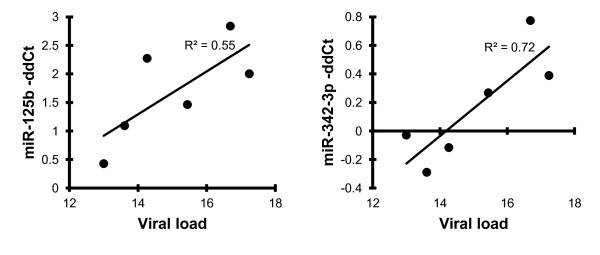
**Correlation of miR-125b and miR-342-3p with viral load**. Correlations were calculated for all miRNAs analyzed by qPCR and for all viremic individuals. The top two correlations by the Pearson method are shown, along with the coefficient of determination. The y-axis units are negative delta-delta Ct (normalized, then related to the mean expression of control samples), which represents relative abundance. Viral load is displayed on a log (2) scale.

Evidence for miRNA-mediated regulation of HIV RNA has previously been presented for several miRNAs, among them miRNAs identified here: miR-29 family members [37], miR-125b and miR-150 [15]. Consistent with a potential regulatory role, each of these miRNAs was expressed at higher levels in PBMC from elite suppressors than in cells from viremic individuals (although it must be noted that even in ES, the levels of 125b and 150 were lower than in controls). In contrast, within the viremic group, there were no negative correlations with viral load. Two positive correlations attained (p < 0.05) or approached (p < 0.1) significance: miR-342-3p and the putative HIV modulator miR-125b (Figure 9).

### CD4+ T-cell count and miRNA expression

In contrast, both negative and positive correlations with CD4 cell counts were found when ES and viremic patients were grouped together (Table 2). miRs-150, -29a, -31, and -31* were significantly (p < 0.05) positively correlated with CD4 counts, while miR-181b was negatively correlated. Three additional miRNAs had negative correlations that approached significance (p < 0.1). When analysis was restricted to elite suppressors, an additional positive correlation with miR-34a was revealed (Table 2). Although correlation analyses with small numbers of samples must be interpreted with appropriate caution, the number and significance of these correlations suggests biological relationships between miRNA expression and HIV disease progression.

### CD4+ T-cell decline does not explain all differential expression of miRNA

The most obvious explanation for the correlation of CD4+ T-cell counts with miRNA expression is the well-characterized population shift of blood cell subsets that occurs during HIV infection. Thus, a hypothetical decline of CD4+ T-cell-specific miRNAs would proceed in proportion with CD4+ T-cell depletion, accompanied by an increase in miRNAs specific to other cell types. However, our data suggest that cell subset decline alone does not explain all of the observed variation. In our study, the highest observed CD4+ T-cell count was eight times greater than the lowest count, but several miRNAs varied more greatly, e.g., by 100 fold (miR-31*). miRs -16 and -150 varied only slightly, but were nonetheless correlated with CD4+ T-cell count--and with opposite signs. Additionally, based on the expression of several miRNAs, even ES with high CD4+ T-cell count closely resemble viremic individuals (Figures 3, 4, 6).

## Discussion

We show for the first time that HIV-1-positive elite suppressors are characterized by a PBMC miRNA profile that in general resembles that of uninfected individuals. However, we also find that ES, on the basis of miRNA expression, are not an entirely homogenous group, and that different mechanisms, shaped or marked by different miRNA expression patterns, underlie elite control in different individuals. We demonstrate further that miRNA expression is closely tied to CD4+ T-cell count in both ES and viremic patients, but that CD4 + T-cell counts do not fully explain the observed differences in miRNA profile. We describe significant differential expression of several miRNAs in control, ES, and viremic patients that have not previously been reported in connection with HIV-1 infection, e.g., miR-31 and its star partner, miR-31*. Confidence in our findings is bolstered by rigorous cross-validation by two profiling platforms, multiple-replicate testing by individual qPCR assays, and the use of multiple data normalization and analysis methods to ensure method-independence of results.

Despite the general proximity to controls, elite suppressors as a group display a pattern of expression for several specific miRNAs (e.g., miR-125b and miR-150) that closely resembles that of viremic patients. This finding gives rise to several hypotheses. The similar levels of some miRNAs in all infected samples may imply that immunologic responses to the presence of even low levels of virus and/or an immunologic imprint in the early phase of infection are sufficient to effect reduced expression of specific miRNAs. Alternatively, it is possible that elite suppressors, even prior to infection, maintain lower baseline levels of these miRNAs in comparison with the general population, and that this is protective or indicative of mechanisms of protection. These hypotheses should be tested in longitudinal studies.

A second major implication of our results for miRs-125b and -150 (as well as -382, although it does not reach significance) has to do with the reported role of these miRNAs in latency and control of viral replication [15]. We find no negative correlation of these miRNAs with viral load of viremic patients. In fact, there is a positive trend for miR-125b and viral load. Although ES may have slightly higher levels of these miRNAs than viremic patients, the difference does not seem to be of sufficient magnitude to explain the profound gap in viral loads. It is of course possible that elite suppressors experience a significant decline of these miRNAs in the global PBMC population, but that a small number of specific cells--perhaps infected cells--experience an increase that keeps viral replication in check.

Conversely, the observed alterations in PBMC miRNA expression cannot be attributed solely to infected cells. For miRNAs that are less abundant in PBMC from viremic patients, downregulation must be due almost entirely to bystander effects. Only a small percentage of PBMC are infected in HIV-positive individuals. Thus, even if specific PBMC miRNAs were absent from infected cells, this difference would be undetectable in the overall PBMC population. For overexpressed miRNAs, as well, bystander effects would have to be invoked unless infected cells upregulated these miRNAs by orders of magnitude. Although possible, miRNA expression differences of hundreds- or thousands-fold are rarely observed *in vivo *or *in vitro*. We conclude that most of the expression differences we observe are the result of host responses to infection, indirect effects of viral products, or both.

In addition to miRNAs with known roles in general HIV-1 infection, we describe several previously unreported associations of miRNAs with HIV-1 infection and progression. For three reasons, we suggest that at least one of these miRNAs, miR-31, has widespread implications for the interplay of HIV and T cell identity in latency and disease progression. First, miR-31 expression differences distinguish cells that influence HIV disease: previous publications consistently report that T-regulatory cells contain lower levels of miR-31 than do other T-cells [38,39]. Second, miR-31 is induced by CCAAT/enhancer binding protein beta (C/EBP beta) [40], specific isoforms of which can either inhibit or stimulate retroviral replication [41,42]. Third, the validated target of miR-31, the special AT-rich binding protein 2 (SATB2) [43], is part of a gene regulatory family needed for differentiation within the T-cell lineage. These observations, along with the consistent and profound downmodulation of miR-31 in PBMC of viremic patients and, to a lesser extent, in cells of some elite suppressors, demand experimentation on how the presence and absence of this miRNA affect T-cell differentiation and activation in the context of HIV infection and latency.

Our data suggest that changes in the levels of miR-31, miR-150, and others in PBMC during infection are the result of regulation and are not entirely (if at all) due to CD4+ T-cell decline. This conclusion is strongly supported by a recent study of miRNA profiles in specific blood cell populations [38]. With the possible exception of miR-125b, miRNAs that are positively correlated with CD4+ T-cell count in our work are not found exclusively in CD4+ T-cells or naïve T-cells and thus could not be expected to decline to the observed extent even with complete CD4+ T-cell depletion. Indeed, some miRNAs that have been found at high levels across PBMC subsets (miR-16) or are even enriched in CD4+ T-cell subsets (miR-181 family members, miR-130b) [38]--and would therefore be expected to decline along with CD4+ T-cells--are, to the contrary, negatively correlated with CD4+ T-cell count. Further work is needed to characterize the effects of HIV and immune responses on miRNA expression in PBMC and specific cell types both in vivo and, as far as possible, in primary culture ex vivo.

Several pioneering studies have already initiated the process of answering these questions, foremost among them the work of Houzet, et al. [25] and Huang, et al. [15]. Building on earlier investigations of HIV and miRNA using HIV-1-transfected or -infected cell lines [16,17], Houzet, et al. reported differential regulation of miRNAs in PBMC of HIV-infected, viremic individuals. The direction of this regulation was primarily downward, consistent with our results. Perhaps most importantly, our groups both observed downregulation of miR-150 and miR-29 family members, which have been characterized as miRNAs with HIV regulatory roles. Although Houzet, et al., observed a greater number of significant differences between controls and infected individuals than we report here, we do not see this difference as a discrepancy, due to the sizes of the respective studies, different study design, and use of different profiling platforms. Instead, the similarities of our finding--especially regarding latency-associated miRNAs--are telling and important, and should prompt additional follow-up studies.

Regarding the Huang, et al. study, we find significant and consistent downregulation of two of five miRNAs (miRs -125b and -150) that these authors found to be associated with control of HIV-1 transcripts in CD4+ T-cells and downregulated in CD4+ T-cells of HIV-1 patients [24]. We also observed that another of these five previously reported miRNAs, miR-382, was downregulated approximately two-fold, but not significantly, in PBMC of both ES and viremic patients (not shown). Interestingly, although the five miRNAs from the Huang, et al. CD4+ T-cell study were reported to be downregulated during differentiation of monocytes to macrophages in a follow-up study [20], another group observed downmodulation of only one of these five, miR-223 [23]. In the future, multiple validation techniques and analysis of larger numbers of samples will help to address the important issue of miRNAs involved in HIV control and latency in specific target cell types.

Of potential clinical importance, this study suggests that miRNA profiles may serve as biomarkers for the identification of ES who could benefit from closer monitoring and/or antiretroviral treatment (ART). Among the ES examined here, three had distinctly lower CD4+ T-cell counts. ES with low CD4+ T-cell counts have been described previously [44-46]; one elite suppressor with a low CD4+ T-cell count developed Kaposi's Sarcoma despite undetectable viral load (46). For some ES with declining CD4+ T-cell counts, ART has been initiated, in some cases with positive CD4 response [44,47]. On the basis of the individual qPCR results presented here, two of the three elite suppressors with low CD4+ T-cell counts are unambiguously clustered with the viremic patients. It will be important to expand our investigations to discover additional correlates of miRNA expression and to determine whether miRNA profiles predict clinical decline, facilitating the decision of when treatment should be initiated in ES with declining CD4+ T-cell counts.

## Conclusion

This study, the first report of miRNA profiles in ES versus viremic patients and uninfected donors, underscores the important role of miRNA in antiviral immunity. That miRNA expression is closely tied to--but not entirely explained by--CD4+ T-cell decline during HIV disease shows that miRNAs are involved in processes reflected by traditional markers of HIV disease progression. Several differentially expressed miRNAs identified here, such as miR-125b and miR-150, have also been described previously and appear to influence control of HIV replication. Our results indicate that this role of miRNA in viral latency and control should be investigated longitudinally and in the context of different host responses to infection. Our data reveal heretofore unreported small RNA correlates of disease and nonprogression, including miR-31 and miR-31*, that we trust will prompt both biomarker development and investigations of novel small RNA-based therapeutic options.

## Materials and methods

### Donors

Eight control, seven elite suppressor, and seven viremic donors contributed blood to this study. Controls were healthy, HIV-1-negative donors. The elite suppressors consistently maintain a viral load below the limit of detection (50 copies/mL) and had a mean CD4+ T-cell count of 1048 cells/ul at the time of donation. No ES was hetero- or homozygous for the CCR5 Δ32 genotype. Viremic individuals were not taking antiretroviral therapy and had mean viral loads and CD4+ T-cell counts of 57,350 copies/mL and 380 cells/ul, respectively.

### PBMC isolation

Peripheral blood mononuclear cells were isolated from fresh blood by Ficoll gradient separation. 1 × 10^7 ^cells were mixed with 600 ul mirVana RNA lysis buffer (Ambion, Austin, Texas, USA) to achieve lysis and inactivate endogenous RNAses. Lysates were frozen at -80°C until RNA purification.

### RNA isolation and quality control

RNA was isolated from all samples in parallel to avoid batch effects and the potential influence of slight differences in RNA isolation [30]. Thawed samples were processed for total RNA isolation using the miRVana miRNA isolation kit (Ambion) and following the manufacturer's protocol. RNA concentrations and purity were assessed by NanoDrop spectrophotometer. Quality control was performed by measuring the expression of several small RNAs, including snRNA U6 and miR-16, with individual TaqMan miRNA assays (Applied Biosystems/Life Technologies, Carlsbad, California, USA).

### NanoString analysis

RNA was diluted to 33 ng/ul and eight control, seven ES, and six viremic samples were submitted to the Johns Hopkins Medical Institutes Deep Sequencing and Microarray Core Facility for further processing by the NanoString nCounter system (NanoString, Seattle, Washington, USA). The NanoString miRNA panel detects 664 endogenous miRNAs (with 654 probes), 82 putative viral miRNAs from nine viruses including HIV-1, five housekeeping transcripts [(actin beta (NM_001101.2), beta-2 microglobulin (NM_004048.2), GAPDH (NM_002046.3), RPL19 (NM_000981.3), and RPLP0 (NM_001002.3)], and six positive and eight negative controls (proprietary spike-in controls). Unlike traditional hybridization microarrays, NanoString does not associate targets with spatial coordinates; instead, the system generates copy numbers of target-specific molecular barcodes attached to detection probes, theoretically eliminating position-dependent effects. Raw data, which are proportional to copy number, were log-transformed and normalized by the quantile method after application of a manufacturer-supplied correction factor for 13 miRNAs. Data were filtered to exclude relatively invariant features (IQR = 0.5) and features below the detection threshold (defined by a cutoff corresponding to approximately one standard deviation above background levels) in at least half of the samples. Using R/Bioconductor and the filtered dataset, linear models for microarray data analysis (limma, [48]) was employed with a contrast matrix for the following comparisons: Control vs. ES, Control vs. Viremic, ES vs. Viremic, Viremic vs. Control and ES, and Control vs. Infected (ES and Viremic). P values were used to rank miRNAs of interest, and correction for multiple comparisons was done by the Bonferroni method. Raw data that were above background (analysis not shown), as well as the corresponding quantile-normalized data, were also imported into MultiExperiment Viewer [49,50]. One-way analysis of variance was performed for the three classes, and samples were hierarchically clustered by Euclidean distance and average linkage. Class prediction methods were applied with BRB-ArrayTools [51].

### TaqMan low-density arrays

The microarray cards used in this study detect 384 features on each of two cards, 'A' and 'B': a negative control, three endogenous small RNA controls (one of them in quadruplicate), and 377 human miRNAs. Reverse transcription and pre-amplification were performed with the manufacturer's reagents (Applied Biosystems). Real time quantitative PCR was performed with an ABI 7900 real-time PCR machine (Johns Hopkins DNA Analysis Facility), and data were collected with the manufacturer's SDS software. RQ Manager software (Applied Biosystems) was used to process the array data. Thresholds, set at 0.2, were checked individually and corrected as necessary. Data processing was done as described previously [6].

### Accessibility of array data

Raw data and sets of quantile-normalized, filtered data from NanoString and TaqMan low-density arrays were deposited with the Gene Expression Omnibus (GEO, [52]) and are accessible as SuperSeries GSE33617.

### Individual RT-qPCR assays

TaqMan miRNA assays (Applied Biosystems) were performed according to the manufacturer's protocol and as previously described [6] with either reverse-transcribed or reverse-transcribed and pre-amplified samples. The small RNAs assayed were miRs -16, -21, -22, -27a, -29a, -29c, -31, -31*, -34a, -125b, -126, -130b, -146a, -146b, -150, -155, -181b, -199a, -221, -223, -342-3p, -1274A, let-7b and let-7g, and small RNAs RNU48 and snRNA U6. Delta-delta Ct (ΔΔCt) analysis was performed, with normalization to the geometric mean of the twelve least variant small RNAs across samples and comparison of each sample with the mean expression of the control samples. Results were not dependent on this normalization method, as established by investigations of alternative normalization methods, including normalization to the spliceosomal small nuclear RNA U6 (snRNA U6, data not shown).

### Data analysis, statistical methods, and figures

Data processing and analysis were conducted using tools from Microsoft Excel, GraphPad Prism (Kruskal-Wallis with Dunn's multiple comparison), XLStat (correlation analyses by Spearman and Pearson, Kruskal-Wallis with Conover-Inman multiple comparison), BRB-ArrayTools (binary tree predictions, class prediction) [51], the MultiExperiment Viewer (TIGR; one-way ANOVA, hierarchical clustering by Pearson correlation and Euclidean distance, self-organizing maps, self-organizing tree algorithms, k-means clustering) [49], and R/BioConductor packages including limma (with Bonferroni correction) [48] and HTqPCR (comparisons with Benjamini-Hochberg correction [53] for multiple comparisons) [54]. Figures and tables were prepared using Microsoft Excel and Word, GraphPad Prism, MultiExperiment Viewer, Adobe Photoshop and Illustrator, BRB-ArrayTools, and HTqPCR.

### Ethics Statement

Approval for all studies was granted by the Johns Hopkins Institutional Review Board. All study participants provided informed consent in writing.

## Competing interests

The authors declare that they have no competing interests.

## Authors' contributions

KWW, JNB, and JEC conceived of and managed the study. JNB obtained samples. JEC provided resources. KWW and AKW performed the experiments. KWW analyzed the data and wrote the manuscript. JNB and JEC contributed to revision of the manuscript. All authors read drafts and approved the final manuscript.
